# Follow-up of Mothers with Suspected Postpartum Depression from Pediatrics Clinics

**DOI:** 10.3389/fped.2017.00212

**Published:** 2017-10-03

**Authors:** Nerissa S. Bauer, Susan Ofner, Amy Pottenger, Aaron E. Carroll, Stephen M. Downs

**Affiliations:** ^1^Section of Children’s Health Services Research, Pediatrics, Indiana University School of Medicine, Indianapolis, IN, United States; ^2^Center for Health Services Research, Regenstrief Institute, Indianapolis, IN, United States; ^3^Biostatistics, Indiana University School of Medicine, Indianapolis, IN, United States; ^4^Section of Pediatric and Adolescent Comparative Effectiveness Research, Pediatrics, Indiana University School of Medicine, Indianapolis, IN, United States

**Keywords:** screening, mental health, medical home, postpartum depression, mothers

## Abstract

**Purpose:**

Pediatric providers are increasingly screening for postpartum depression (PD), yet, it is unknown how often mothers comply with recommendations to seek treatment. The objectives were to describe the rate at which mothers with suspected PD seek treatment and explore factors that predict help-seeking behavior.

**Design and methods:**

Mothers were recruited from four pediatric clinics after identification using the Child Health Improvement through Computer Automation (CHICA) system. Mothers with a positive screen were invited to participate in a telephone interview between January 2012 and December 2014. Mothers reported if they sought treatment or called a community resource.

**Results:**

73 of 133 eligible mothers participated (55% response rate). Fifty women recalled a recommendation to seek help. Only 43.8% (32/73) made a follow-up appointment with an adult provider and even fewer kept the appointment.

**Conclusion:**

A majority of mothers suspected of having PD recalled a referral for further intervention; yet, less than half took action. Further investigation of barriers of help-seeking behavior is warranted.

## Introduction

Postpartum depression (PD) is a common and disabling mental health (MH) condition that affects approximately 15% of women after childbirth (1). Prevalence estimates are even higher among women from lower socioeconomic strata and ethnic minorities (2–4). When PD occurs, it is most common when the newborn infant is between 6 weeks and 6 months of life; however, it can occur anytime within the first year postpartum (5). Strong evidence associating PD with adverse childhood outcomes related to growth, development, and subsequent risk for MH and behavioral conditions supports pediatric providers’ routine screening for PD and other forms of parental depression (6–10). Exposure to parental depression can also impair parenting practices, parent–child interactions, and children’s functioning at home and school (11–18).

To date, much of the efforts around PD within the field of pediatrics have focused on increasing pediatric providers’ awareness and promoting screening in routine practice. Clinical care guidelines published by the American Academy of Pediatrics (AAP) support pediatric providers’ active role in implementing surveillance and systemic screening in practice (19). Several validated and brief screening tools exist to aid providers’ efforts to screen during well child visits (20–23). However, studies show that rates of screening for PD remain less than optimal (24–26). Furthermore, when mothers with depression are open to seeking help and able to access adequate MH treatment, children’s behavior and functioning improve (27). Unlike obstetricians who can both screen and treat the mother for PD (28, 29), pediatric providers find themselves in a challenging position because they can identify PD, but can only facilitate getting mothers into treatment by adult providers. Moreover, women with PD historically have exhibited low rates of help seeking behavior (30, 31). While there have been limited studies identifying treatment-seeking behavior among identified mothers (32), there are no studies examining the moment of a positive screen and the communication that occurs between pediatric provider and the mother. There are no studies describing help-seeking rates of mothers suspected of having PD after identification by pediatric providers. It is unclear whether pediatric providers are effective in helping mothers take action concerning their own health.

The objectives of this study were twofold: (1) to describe the rate at which mothers suspected of having PD sought treatment after a positive screen occurred in a pediatric practice and (2) to explore factors associated with mothers seeking treatment. Understanding what factors are associated with mothers having a more positive attitude toward seeking treatment, such as modifiable aspects of provider–mother communication during the screening process, may help optimize the interaction between the pediatric provider and mother at the time of a positive screen.

## Materials and Methods

### Study Population and Design

Participants included mothers whose children received medical care at one of the four general pediatrics clinics and were either suspected of having PD as a result of a positive screen and/or referred for an evaluation for PD at any time within the first 2 years of the child’s life. Families who receive care at these clinics are largely underserved and of lower socioeconomic status who are eligible to enroll in Medicaid.

Eligible mothers were identified through the Child Health Improvement through Computer Automation (CHICA) system (33), a computer decision support system that is linked to the electronic health record and described in detail elsewhere (34–38). Briefly, CHICA is used to automate surveillance and screening of preventive care and chronic conditions based on clinical care guidelines (39–43). For PD, CHICA performs surveillance every 3 months starting at birth and up to 15 months of age (44). CHICA generates the anxiety subscale of the Edinburgh Postpartum Depression Scale (EPDS-3), which has previously been shown to have a high sensitivity (95%) and negative predictive value (98%) for PD, to pre-screen families in the waiting room at the clinic (22). The EPDS-3 items focus on whether the mother blames herself unnecessarily when things go wrong, feeling scared, or panicky for no good reason, and being anxious or worried for no good reason. These items are integrated within a tailored 20-item pre-screener form that is generated based on the child’s age and information contained in the EHR (45, 46). This form is displayed electronically on a tablet that is given to the parent upon check in and is completed in the waiting room (47). If any of the EPDS-3 items is positive, CHICA generates the entire 10-item EPDS (20) to screen the mother for PD formally. Additionally, CHICA generates a handout for the provider to share with the mother. It describes PD and suggests community resources. Providers document on another CHICA-generated form, the physician worksheet, whether they suspect PD and/or have referred mothers for treatment of PD after review of the EPDS. Mothers were eligible for this study if the provider marked the checkbox indicating a recommendation was made to the mother to seek additional help and/or the checkbox indicating suspected PD. During the study period, a monthly data pull of all eligible mothers was provided to the study team.

Within a few days of the data pull, eligible mothers were mailed a study information sheet and letter explaining the study and alerting her that a research assistant would call after 2 weeks to invite the mother to complete a one-time, 20-min telephone interview. Mothers could call to schedule the interview or opt out of the study. Informed consent was obtained verbally over the phone at the time of the interview, and interview questions were read in English or Spanish. If, during the interview, a mother expressed worries of harming herself or her child, an emergency protocol was initiated: the research assistant provided the mother with a phone number to call for help and the child’s provider was notified immediately.

The primary objective of the study was to describe the rate at which mothers sought treatment after a positive PD screen and to explore factors that would predict mothers’ help seeking behavior. “Seeking treatment” was broadly defined as a mother reporting either of the following: (1) the making an appointment with a primary care physician (PCP), obstetrician, or MH provider; or (2) contacting any community resource from a CHICA-generated handout regarding PD and a listing of local resources.

### Telephone Interview Tool

Mothers consenting to the study were asked to answer a 42-item telephone survey. The survey included study-specific items (whether the mother remembered completing a formal screening tool for her mood, what term the provider used when talking about her mood, if she received a list of community resources to go to or call for help, whether she received recommendations to see an adult provider and if so, what type of provider, and what actions she took after the positive PD screen). Additional items came from validated tools to understand whether continuity of care and degree of family centeredness (select items from the Promoting Healthy Development Survey/PHDS (48) and degree of shared decision-making (Shared Decision-Making Questionnaire/SDM-Q-9) (49)) were associated with help-seeking behavior. The full-scale PHDS was shown to have strong construct validity (mean factor loading: 0.69) and internal consistency (Cronbach’s α: 0.80) (50). The SDM-Q-9 measures the level of shared decision-making during the medical encounter and has been shown to have high internal consistency (Cronbach’s α > 0.9) and high item discriminations for high reliability of the instrument in a primary care sample (49). Items are rated on a 6-point Likert scale from 0 = completely disagree to 5 = completely agree. Seven of nine items must be completed to score the scale. Items were added to calculate a raw score (0–45), which was then transformed to a range of 0–100 by multiplying the raw score by 20/9. Higher scores indicated increased shared decision-making between pediatrician and mother. When mothers did not seek help, reasons for not taking action were asked. Sociodemographics and characteristics (first child, race and ethnicity, maternal age, highest education level, marital status, and the number of children under 18 years of age in the household) were also captured. The tool was translated into Spanish and back translated to English to assure retention of meaning.

### Statistical Analysis

Demographic and other subject characteristics were summarized overall and by the primary outcome of seeking treatment (yes/no) and by the secondary outcome of the child’s doctor giving the mother a recommendation for follow-up with her primary doctor, an obstetrician, or a MH provider. Characteristics were compared by means of chi-square and Fisher’s exact test for categorical variables and by one-way analysis of variable models for continuous variables. Though the sample size was small, we decided to explore associations between outcomes and demographic characteristics (age, first child, race, Hispanic ethnicity, and marital status), the physician saying “postpartum depression,” predictors of family centeredness, and shared decision-making. We fit bivariate logistic regression models with the independent variables recoded as binary with agree/disagree response categories so as to facilitate the interpretation of odds ratios. Prior to fitting multivariable logistic regression models, we assessed whether we had enough data. Using Hosmer and Lemeshow’s (51) extension to multiple regression models of Peduzzi’s (52) result that the number of parameters in the model should be limited by the smaller of the number of events or non-events divided by 10, we found that we could have three variables in the primary outcome model and two variables in the secondary outcome model. We chose clinically important variables as eligible for inclusion: being a teen mother (aged <21 years), Hispanic, black race, other race, being married, being separated, the SDM-Q-9 composite score, and the PHDS quality score. Items from the SDM-Q-9 scale were also tested. Variables with *p*-value of 0.30 or smaller in bivariate models were selected for inclusion in a multiple regression model. Backwards variables with staying criteria of 0.10 were used to come up with final multivariable logistic regression models. To explore the relationship between the two outcomes, we fit the dependent variable of seeking treatment and independent variable of the doctor giving the mother a recommendation for follow-up. The study protocol was submitted to our institutional review board and approved prior to the initiation of study procedures. Analyses were generated using SAS/STAT software, Version 9.4 of the SAS System for Windows (copyright © 2002–2008 SAS Institute Inc.).

## Results

Of 133 mothers found to be eligible, 73 mothers agreed to participate in the telephone interview and had adequate information to be analyzed. Fifty-three women could not be reached, either due to bad phone numbers (46 women), or a refusal to participate (7 women). Only one had missing data regarding which type of adult provider was recommended, but she did report making an appointment; eight had completely missing data for “seeking treatment” and thus were excluded in the multivariable analyses. The average age among mothers was 26.8 years, with a SD of 6 years. Most of the mothers (41% or 30/73) were African American, 21% (15/73) white, and 8% (6/73) mixed race. Just under half (45.2 or 33/73) were non-Hispanic. Sixty-six percent were never married and 40% (29/73) had not completed a high school education. Approximately one-third of the mothers were identified after a positive screen with their first child. The average age of the child at the time of the telephone interview was 9.2 months, with a SD of 6 months. More than half of the women reported depressive symptoms for more than 2 weeks at a time since the child was born. Forty-eight percent (35/73) of women reported having felt sad or depressed most days for 2 years or more. These and other data are summarized in Table 1.

**Table 1:** Study sample characteristics.

		Overall
		*n* = 73
Age	*N*	73
	Mean ± SD	26.8 ± 6.0
	Median (min, max)	25.0 (17.0, 44.0)

Hispanic or Latino origin	Missing	4
	Yes	33 (47.8)
	No	36 (52.2)

Race	Missing	2
	White	15 (21.1)
	African American	30 (42.3)
	Native American	1 (1.4)
	Native Hawaiian	1 (1.4)
	Other	18 (25.4)
	Mixed race	6 (8.5)

Education: highest grade level of school completed	Missing	1
	Less than high school	29 (40.3)
	High school	25 (34.7)
	Some college	18 (25.0)

Marital status	Married	16 (21.9)
	Separated	9 (12.3)
	Never been married	48 (65.8)

First child	Yes	26 (35.6)
	No	47 (64.4)

Childs age (in months)	N	73
	Mean ± SD	9.2 ± 6.0
	Median (min, max)	9.0 (1.0, 24.0)

Felt sad, blue, or depressed for 2 weeks or more since child was born	Yes	40 (54.8)
	No	33 (45.2)

Had 2 or more years when felt depressed or sad most days	Yes	35 (47.9)
	No	38 (52.1)

**Figure 1:**
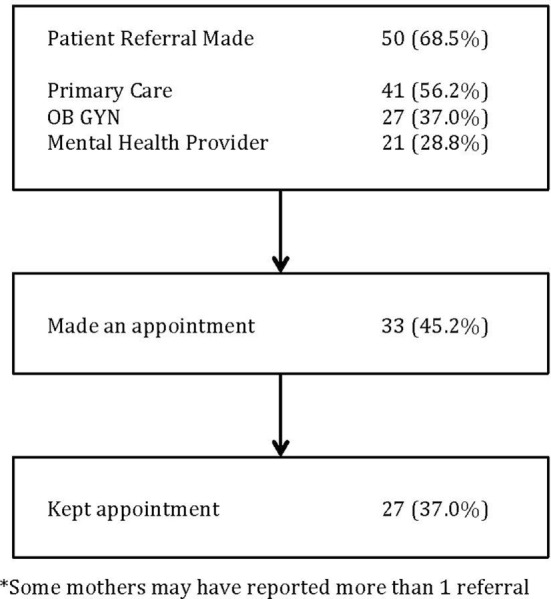
Actions taken by mothers after a positive PD screen (*N*=73)*. *Some mothers may have reported more than one referral.

Approximately two-thirds of the women (50/73 or 68.5%) reported that their pediatric providers recommended further evaluation by an adult care provider. Of those, 46 were also provided a handout with community resources. About half (36/73) took actions that were defined as “sought treatment” after the index visit, one-third (22/73) called any type of adult provider and made an appointment, 13.7% (10/73) made an appointment and called any community resource from the handout, and 5.5% (4/73) only called a community resource. An even smaller proportion of mothers actually made it to the scheduled appointment with an adult provider (37% or 27/73). See Figure 1. Nineteen percent (14/73) of mothers recalled only receiving a handout and 8% (6/73) called any of the resources listed.

Mothers had various reasons for not following through with recommendations, which included a perception that mood would improve (*n* = 3); lack of time or competing demands that necessitated mothers’ assigning other duties as more important (*n* = 7) or transportation (*n* = 2). Others mentioned that they were already seeing a social worker or therapist (*n* = 3), and others reported frustration with navigating the telephone tree (*n* = 2) or expecting the call to come from the clinic (*n* = 1).

Maternal demographics did not significantly predict whether a mother received a recommendation for further help nor did they predict whether a mother ultimately took actions to seek help. Several provider behaviors were recalled significantly more often by mothers who received advice to seek further help from an adult provider as compared to mothers who did not recall this advice: provider used the term “postpartum depression” (56% vs. 18%, *p* = 0.005) and exhibited more of a shared decision-making style (mean SDM-Q-9 score: 66.8 ± 24.0 vs. 47.1 ± 31.1, *p* = 0.03). More specifically, the behaviors that were significant were: the provider made explicit that a decision needed to be made about her health (*p* < 0.0001), explained that there were different options for treatment of her mood (*p* = 0.004), and provided explanation of the advantages and disadvantages of each (*p* = 0.01). See Table 2.

**Table 2:** Bivariate summaries and odds ratios for mothers who recalled recommendation for treatment.

		Recommendation				Sought treatment			
		Yes	No	*p*-Value	Odds ratio (95% confidence interval)	Yes	No	*p*-Value	Odds ratio (95% confidence interval)
	
		50 (69.44%)	22 (30.56%)			42 (64.6%)	23 (35.4%)		
Age <21		6 (12.0)	4 (18.2)	0.4875	0.61 (0.16, 2.44)	7 (16.7)	3 (13.0)	0.6993	1.33 (0.31, 5.74)
First child		16 (32.0)	9 (40.9)	0.4656	0.68 (0.24, 1.92)	14 (33.3)	10 (43.5)	0.4189	0.65 (0.23, 1.85)
Hispanic		24 (49.0)	9 (47.4)	0.9051	1.07 (0.37, 3.08)	19 (47.5)	9 (40.9)	0.6182	1.31 (0.46, 3.74)

Race	Black	19 (39.6)	10 (45.5)	0.7186	1.27 (0.35, 4.58)	17 (41.5)	10 (45.5)	0.9335	0.94 (0.25, 3.62)
	Other	20 (41.7)	6 (27.3)	0.2561	2.22 (0.56, 8.82)	15 (36.6)	7 (31.8)	0.8091	1.19 (0.29, 4.90)
	White	9 (18.8)	6 (27.3)		Reference	9 (22.0)	5 (22.7)		Reference

Marital status	Married	11 (22.0)	5 (22.7)	0.9606	1.03 (0.30, 3.50)	7 (16.7)	8 (34.8)	0.0905	0.35 (0.10, 1.18)
	Separated	7 (14.0)	2 (9.1)	0.5654	1.64 (0.30, 8.86)	5 (11.9)	3 (13.0)	0.6151	0.67 (0.14, 3.24)
	Never been married	32 (64.0)	15 (68.2)			30 (71.4)	12 (52.2)		Reference

Felt sad, blue, or depressed for 2 weeks or more since child was born		29 (58.0)	11 (50.0)	0.5298	1.38 (0.51, 3.78)	24 (57.1)	11 (47.8)	0.4721	1.46 (0.52, 4.04)

Child’s doctor said “postpartum depression”		28 (56.0)	4 (18.2)	0.0050	**5.73 (1.69, 19.37)**	23 (54.8)	8 (34.8)	0.1265	2.27 (0.79, 6.50)

PHDS quality	Mean ± SD	92.9 ± 20.8	93.2 ± 19.7	0.9620	1.00^a^ (0.88, 1.13)	95.6 ± 16.6	92.8 ± 21.8	0.5630	1.04^a^ (0.91, 1.19)

SDM9 composite	Mean ± SD	66.8 ± 24.0	47.1 ± 31.1	0.0272	**1.15 (1.02, 1.29)**	68.0 ± 25.6	61.9 ± 24.7	0.4197	1.05^a^ (0.94, 1.18)

Make it clear that a decision about mother’s health needs to be made		44 (89.8)	4 (30.8)	0.0001	**19.80 (4.43, 88.52)**	34 (85.0)	14 (77.8)	0.5029	1.62 (0.40, 6.63)

Asks how mother wants to be involved in decision-making		41 (87.2)	9 (64.3)	0.0598	3.80 (0.95, 15.23)	35 (92.1)	15 (75.0)	0.0867	3.89 (0.82, 18.39)

Explains different options for treating mother’s mood		42 (89.4)	8 (53.3)	0.0044	**7.35 (1.86, 29.05)**	35 (87.5)	15 (83.3)	0.6713	1.40 (0.30, 6.62)

Explains advantages or disadvantages of the treatment options		30 (69.8)	5 (31.3)	0.0103	**5.08 (1.47, 17.57)**	26 (70.3)	10 (58.8)	0.4092	1.66 (0.50, 5.47)

Helps her understand all the information		44 (93.6)	15 (78.9)	0.0964	3.91 (0.78, 19.52)	37 (92.5)	19 (90.5)	0.7847	1.30 (0.20, 8.45)

Together choses treatment option		27 (62.8)	7 (50.0)	0.3992	1.69 (0.50, 5.70)	22 (64.7)	12 (66.7)	0.8876	0.92 (0.27, 3.06)

Agrees with her on how to get help		34 (77.3)	9 (50.0)	0.039	**3.40 (1.06, 10.87)**	28 (77.8)	14 (66.7)	0.3608	1.75 (0.53, 5.81)

*^a^For 5% increase. Multivariable modeling of help-seeking behavior resulted in only one statistically significant explanatory variable; namely, if the mother reported the provider simply having the discussion after a positive screen (72 vs. 22.7%; AOR: 4.63; 95% CI: 1.32–16.24)*. *Significance of bold font are results that are statistically significant and for ease of review*.

## Discussion

A majority of women suspected of having PD after an initial positive screen in the pediatric office recalled a discussion with the pediatric provider about the need to seek follow-up with an adult provider. However, successively smaller proportions of those mothers took steps to seek treatment for themselves. Maternal demographics did not predict whether mothers recalled receiving a recommendation for further treatment when the pediatric provider shared the results of screening or in eventually seeking treatment.

While it is known many mothers with PD do not seek help, our study is the first to examine what happens after a positive PD screen. The number of pediatric providers screening for PD are increasing (53); however, our study highlights that sharing the results of screening and what is communicated to the mother can impact whether she seeks help. Our study also suggests a shared decision-making approach may be helpful in getting mothers to consider taking action. In particular, explaining what treatment options are available and the benefits and disadvantages of each in the context of the importance of seeking help. Our work complements studies examining communication factors and preferences for depression advice within obstetrics and gynecology clinics examining management after a positive screen (54, 55). However, while provider communication can be modified, it is clear that more work is needed in examining additional methods to get mothers linked to active treatment. Closer examination of maternal barriers should include the influence of personally held beliefs and attitudes, cultural and societal norms, and access to resources. In a qualitative study by Sword et al., mothers described discomfort with discussing MH concerns, fears, and deciding to wait for symptom improvement as reasons to defer care (56). Mothers with limited understanding of PD or who had friends or family who normalized symptoms were less likely to seek treatment (56, 57). In yet another study among underserved Hispanic women, only half of the women with a positive PD screen recognized a need to seek help for depression (58). Tailoring health messages about signs and symptoms of PD for at-risk women is a necessary component of future interventions; otherwise, it would be difficult to mobilize women to seek treatment if they lacked insight into the need for it. Furthermore, mothers may fear that disclosing symptoms will lead to child protective services involvement (59). Women in our sample endorsed perceptions that their mood would improve with time, along with resource or material barriers, such as lack of time and childcare issues or difficulty with navigating the telephone appointment system, which have been also been reported as barriers to help seeking in other studies (60). Alternatively, facilitators of help-seeking behaviors should also be considered. Among women seen in a hospital-based obstetrics and gynecology clinic, factors associated with help seeking included if the mother identified the obstetrician/gynecologist as their primary care provider, had previously used MH care, had at least one chronic health condition, or had high depressive symptoms (55). Thus, the development of primary care-based interventions should strive to take into account the myriad of intrinsic barriers, as well as facilitators toward seeking care in order to be effective. It also raises the need for pediatricians to partner with and include the help of pediatric nurse practitioners, nurses, and social workers in the pediatric medical home at the time a positive screen occurs.

Pediatricians’ attitudes and practices around screening for PD are additional targets for optimizing outcomes for mothers suspected of PD (25). Pediatricians who rely solely upon observational cues may under-identify mothers at-risk for PD (61, 62). Additionally, studies have uncovered differences in providers’ and women’s cues to identify depression (58). Further, the course of depressive symptoms across the first year postpartum has high variability (63). It is likely that screening done only once in the postnatal period may not be enough to detect women whose symptoms persist beyond the first 3 months or develop later. Currently, the AAP encourages pediatricians to screen at the 1, 2, 4, and 6-month well child visits (19); however, data suggest that adherence to recommended screening guidelines is low (53, 64). Similar efforts to boost pediatricians screening around developmental delays, including clinical care guidelines for developmental screening during well child visits have yielded improvements in clinicians’ screening rates, but remain less than optimal (65). Thus, integrating innovative ways to increase systems-level screening, such as through automation and health information technology as we have done with our work using CHICA (41) should be ongoing.

The index visit where a positive PD screen occurs should be viewed as a “teachable moment.” This study suggests that if the pediatric provider employs effective communication strategies, it may facilitate help-seeking behavior from mothers suspected of having PD. While communication factors are broad, in one study among Latino immigrants, simply having someone (such as a family member, friend, neighbor, minister) alert them to having an emotional problem was associated with help seeking (66). Although more studies are needed to elucidate mechanisms underlying communication and health outcomes, one review suggested that women who feel comfortable with and have an established relationship with a primary care provider, coupled with ensuring patient understanding, trust, and clinician–patient agreement may lead to adherence and better self-care skills (67). However, known provider-level barriers include lack of training, low self-confidence on how to begin the conversation, what potential actions to take based on mothers’ depression severity, or where to refer in the event of a positive screen (68). The use of practical and high yield communication strategies to handle sensitive, psychosocial topics in primary care, such as the common factors approach (69) or motivational interviewing, may allow the pediatrician to capitalize upon the “teachable moment” and spur mothers to seek help (70). Yet, modifying provider communication may not be enough and more multimodal strategies are needed. Stepped care or medical home models to foster collaboration between pediatricians and co-located MH providers may improve access to treatment rates for mothers with suspected PD (71, 72). While not limited to PD, the “Motivating Our Mothers (MOM) pilot” consisted of screening mothers of children 0–12 years of age for depression during well child visits and providing mothers with written and verbal targeted depression education and motivational messages for help-seeking (73). However, more work is needed to examine whether providers’ communication styles and practice models ultimately lead to improved outcomes through activation of mothers to seek help or other mechanisms.

As with all studies, there are inherent limitations in this work that warrant consideration. First, this was an observational study comprised of a small sample recruited from community clinics in the Midwest. Of 133 eligible women, 73 consented to complete the interview, for a participation rate of 55%. As per our usual study protocol, each eligible subject receives an average of three contact attempts (range 1–10). Therefore, participation was limited by the use of a convenience sample from those reached by phone, which may bias results. Moreover, state-level and institutional policies may have influenced practice patterns related to screening, and thus our results may not be generalizable in other areas. Second, we relied upon maternal report of events, which are subject both to social desirability and recall bias. Some might argue that mothers did not actually seek treatment based on our definition; however, it is important to understand what steps mothers take to help themselves. In this study, the measurement of seeking treatment was either calling to make an appointment or calling a community resource; however, in order to impact outcomes, mothers who actually keep appointments and engage with MH are more likely to have improvements that impact outcomes (27). It is encouraging that among the mothers who reported making an appointment, a high proportion of them actually reported attending the appointment. This is similar to other work with Chinese adults with MH problems that showed intention and behavior change are correlated (74). Similarly, willingness to seek professional help for a serious emotional problem and feeling comfortable talking about personal problems have also been shown to be associated with future help seeking and treatment use from a large cohort of respondents in the US National Comorbidity Survey (75). Therefore, using the teachable moment to raise mothers’ intention to seek help may be powerful. Understanding each step toward getting mothers to access treatment identifies potential targets for intervention that may ultimately improve the pediatric medical home to support mothers and their infants. Last, our study included mothers who were screened based upon CHICA’s clinical care algorithm, which includes PD screening through 15 months of age. While the age range extends screening beyond the recommended first 6 months of life (19), studies have shown that while a majority of PD occurs within the first 3 months post-delivery, some women may continue to develop symptoms of PD through the first year (76, 77). Mothers with a prior history of PD or major depression are at highest risk for recurring symptoms in the first year (78). Moreover, the decision to screen for an additional 3 months beyond 1 year was because our families may not always make it to the clinic for the 12-month well-child visit. The choice to increase the age range at which CHICA automatically screens were thus intended serve as a safety net.

## Conclusion

Postpartum depression is common. Pediatric providers acknowledge that there is a need to screen and identify mothers at-risk as untreated PD leads to negative outcomes for the child and parent–child dyad. Mothers who receive clear communication from the providers about suspected PD and the need to seek treatment from an adult provider are more likely to take the first step toward getting help. However, more work is needed to tailor interventions to take into account the systems-level and maternal-level barriers likely contributing to mothers currently not seeking treatment.

## Ethics Statement

This study was carried out in accordance with the recommendations of our institutional review board and approved prior to the initiation of study procedures. Subjects were consented by telephone and were mailed a copy of the study information sheet and informed consent document as approved by our institutional review board.

## Author Contributions

NB is the first author and responsible for the conception and design, analysis, and interpretation of the data. NB developed the telephone interview and script, emergency protocols, and trained the research assistants. She drafted the article and revised it critically with the input of co-authors and other individuals who agreed to read early versions of the manuscript. SO is a biostatistician who completed the data analysis and worked with NB on interpretation of final results. She is a co-author who helped with the initial draft of the statistical methods and results section and helped with the revision of the entire manuscript prior to submission. AP is a research assistant and lead study coordinator for the project. She oversaw study protocol implementation, assisted NB with human subjects, conducted telephone interviews, and involved with data collection and data entry. She conducted a literature review and revised the manuscript prior to submission. AC is a co-author who helped with the revision of the entire manuscript. SD is a co-author and has provided mentoring and support to NB, provided input into study design, and revised paper.

## Conflict of Interest Statement

The authors declare that the research was conducted in the absence of any commercial or financial relationships that could be construed as a potential conflict of interest.

## Abbreviations

PD, postpartum depression; CHICA, child health improvement through computer automation; EHR, electronic health record; PCP, primary care physician; MH, mental health.
